# Plasma Neurofilament Light Chain and Phosphorylated Tau Are Elevated in Myotonic Dystrophy Type 1

**DOI:** 10.3390/jcm14228197

**Published:** 2025-11-19

**Authors:** Masanori P. Takahashi, Harutsugu Tatebe, Hiroto Takada, Takahiro Nakayama, Michio Kobayashi, Kosuke Yoshida, Satoshi Kuru, Natsuki Kira, Tomoya Kubota, Yasuaki Mizutani, Hirohisa Watanabe, Yuhei Takado, Takahiko Tokuda

**Affiliations:** 1 Clinical Neurophysiology, Department of Clinical Laboratory and Biomedical Sciences, The University of Osaka Graduate School of Medicine, Yamadaoka, Suita 565-0871, Osaka, Japankubota.tomoya.sahs.med@osaka-u.ac.jp (T.K.); 2 United Graduate School of Child Development, The University of Osaka, Yamadaoka, Suita 565-0871, Osaka, Japan; 3 Institute for Quantum Medical Science, National Institutes for Quantum Science and Technology, Chiba 263-8555, Japan; 4 Department of Neurology, NHO Aomori National Hospital, Aomori 038-1331, Japan; takada.hiroto.yw@mail.hosp.go.jp; 5 Department of Neurology, Division of Neuromuscular Diseases, Yokohama Rosai Hospital, Yokohama 222-0036, Kanagawa, Japan; tnakayama.yrh.neuro@yokohamah.johas.go.jp; 6 Department of Neurology, NHO Akita National Hospital, Yurihonjo 018-1301, Akita, Japan; kobayashi.michio.cp@mail.hosp.go.jp; 7 Department of Neurology, NHO Asahikawa Medical Center, Asahikawa 070-8644, Hokkaido, Japan; koyoshida-hok@umin.ac.jp; 8 Department of Neurology, NHO Suzuka National Hospital, Suzuka 513-0004, Mie, Japan; kuru.satoshi.rb@mail.hosp.go.jp; 9 Department of Neurology, Fujita Health University School of Medicine, Toyoake 470-1192, Aichi, Japanhirohisa.watanabe@fujita-hu.ac.jp (H.W.); 10 Institute for Quantum Life Science, National Institutes for Quantum Science and Technology, Chiba 263-8555, Japan; takado.yuhei@qst.go.jp; 11 Department of Neuroetiology and Diagnostic Science, Healthy Longevity Medicine, Osaka Metropolitan University Graduate School of Medicine, Suita 545-8585, Osaka, Japan

**Keywords:** myotonic dystrophy, biomarker, blood, amyloid-beta, neurofilament light chain, phosphorylated tau

## Abstract

**Background/Objectives**: Myotonic dystrophy type 1 (DM1) is a multisystem disorder that affects the central nervous system. Despite previous studies, blood-based biomarkers have not been sufficiently characterized. This study investigated plasma neurofilament light chain (NfL), phosphorylated tau (p-tau181), amyloid-β (Aβ42/40), and glial fibrillary acidic protein (GFAP) in a Japanese cohort with DM1 to assess their potential as biomarkers. **Methods**: Forty patients with genetically confirmed DM1 were enrolled in this study. Plasma NfL, p-tau181, Aβ42/40, and GFAP were quantified using single-molecule array technology. Clinical and genetic variables, including age, CTG repeat size, Mini-Mental State Examination (MMSE) score, modified Rankin Scale (mRS) score, and creatine kinase levels, were analyzed for correlations. **Results**: NfL and p-tau181 were significantly elevated in patients with DM1 compared with controls, with 95% exceeding the p-tau181 cut-off. NfL was moderately correlated with age, age at onset, and mRS, and no significant associations were observed between p-tau181 and other biomarkers, although a correlation was noted with serum creatine kinase. **Conclusions**: These findings support that NfL is a marker of disease severity in DM1 and identified plasma p-tau181 as a potential novel biomarker. While the mechanisms underlying the increased p-tau181 levels remain unclear, they may reflect DM1-related pathologies in the brain and possibly in skeletal muscle. Study limitations include a small sample size and lack of age-matched controls, highlighting the need for longitudinal validation. This study demonstrates the utility of NfL and suggests that p-tau181 is an emerging biomarker for DM1, supporting future work toward biomarker-guided monitoring and therapeutic evaluation.

## 1. Introduction

Myotonic dystrophy (DM), the most common hereditary muscle disease among adults and represents a multisystemic disorder extending far beyond the skeletal muscle. It is caused by an unstable expansion of a CTG trinucleotide repeat in the 3′ untranslated region of the *DMPK* gene, leading to the accumulation of toxic RNA transcripts in the nucleus [1,2]. These transcripts form RNA foci that sequester RNA-binding proteins, such as members of the MBNL family, thereby disrupting the regulation of alternative splicing and other RNA processing events. The resulting spliceopathy affects multiple tissues and explains the systemic nature of the disease [3]. Clinically, DM1 is characterized by progressive muscle wasting and myotonia, frequently accompanied with cataracts, cardiac conduction defects, insulin resistance, dyslipidemia, tumors, and central nervous system (CNS) abnormalities [1,4,5].

Among the affected systems, the CNS’s involvement in DM has been recognized as a major contributor to disease burden; however, its objective evaluation remains challenging [6,7]. The clinical manifestations are heterogeneous, encompassing cognitive impairment, apathy, daytime sleepiness, and personality changes [8], but their severity, and pattern vary widely among individuals [6]. Neuropsychological assessments have revealed deficits in executive function, attention, and visuospatial processing, yet the overlap between cognitive impairment and motivational or attentional disturbances often complicates interpretation [9,10]. Brain imaging studies have revealed diffuse structural and functional alterations in the brain, most frequently in the frontal and temporal regions, but without clear region-specificity or correlation with clinical severity [11,12,13]. Likewise, neuropathological examinations have not identified consistent lesions comparable to those in classical neurodegenerative diseases such as Alzheimer’s disease (AD) (e.g., senile plaques) and Parkinson’s disease (e.g., Lewy bodies), Instead, they demonstrate mild, nonspecific changes, including neuronal loss, gliosis, and white matter abnormalities, the clinical significance of which remains uncertain [14]. Collectively, these limitations underscore the difficulty of quantifying CNS dysfunction in DM1 and highlight the need for objective biomarkers that can sensitively capture the underlying pathology.

At the molecular level, CNS involvement in DM1 is thought to arise from the toxic accumulation of expanded CUG-repeat RNA transcripts in the nucleus, which sequester RNA-binding proteins such as MBNL and alter the activity of CELF1. This imbalance leads to widespread splicing dysregulation across many neuronal genes, including *APP*, *GRIN1* (encoding an NMDA receptor subunit), *CAMK2B*, and *MAPT* (*encoding microtubule-associated protein tau*) [3,15,16,17]. Such abnormalities affect synaptic transmission, calcium signaling, and cytoskeletal dynamics, providing a mechanistic basis for the observed neurobehavioral symptoms [18]. Notably, neurofibrillary tangles in DM1 have been found to contain mis-spliced tau protein, linking the disease to both a spliceopathy and a tauopathy [19].

Distinct conformational and biochemical patterns of tau aggregation define the major tauopathies. In Alzheimer’s disease (AD), tau inclusions consist of a mixture of both 3-repeat (3R) and 4-repeat (4R) isoforms, whereas progressive supranuclear palsy (PSP) and Pick’s disease are characterized predominantly by 4R and 3R tau, respectively [20]. These isoforms arise from alternative splicing of several exons within the *MAPT* gene, most notably exon 10, whose inclusion or exclusion yields the 4R and 3R isoforms. In the healthy adult brain, this splicing event is tightly regulated to maintain an approximately 1:1 ratio of 3R and 4R tau, ensuring proper microtubule stability and axonal transport [21]. The selective aggregation of specific tau isoforms in each tauopathy is thought to play a central role in disease-specific pathology and neurodegeneration. In myotonic dystrophy type 1 (DM1), however, aberrant MAPT splicing has been documented at multiple exons—including exon 10 as well as exons 2/3 and 6—resulting in an altered 3R/4R ratio, often with a relative predominance of the 3R isoform. This disequilibrium may impair tau–microtubule interactions and promote abnormal tau accumulation, thereby contributing to neuronal dysfunction and central nervous system pathology in DM1 [19].

It has been demonstrated that fluid biomarkers, such as those in cerebrospinal fluid (CSF) and plasma, play a crucial role in the diagnosis prognostication, and biological staging of AD [22]. These biomarkers have transformed the conceptual framework of neurodegenerative disorders by enabling in vivo assessment of molecular pathology [23]. In recent years, advancements in micro-measurement technologies, such as single-molecule array (Simoa) assays, have greatly enhanced the feasibility of plasma biomarkers [24]. Because plasma sampling is minimally invasive and suitable for repeated measurements, it has garnered increasing attention as a practical alternative to CSF analysis [25].

Among the major analytes investigated, amyloid β (Aβ), tau, glial fibrillary acidic protein (GFAP), and phosphorylated tau (pTau) have been recognized as key markers reflecting distinct aspects of CNS pathology. Among these, tau and pTau are considered indicative of AD-like tau phosphorylation and aggregation processes [26], GFAP reflects astrocyte activation and gliosis, while neurofilament light chain (NfL) reflects the extent of axonal degeneration and overall neuroaxonal injury [27,28]. Notably, NfL elevation is not specific to AD but has also been reported in a wide spectrum of neurodegenerative conditions, including Parkinson’s disease-related disorders, frontotemporal lobar degeneration, and amyotrophic lateral sclerosis (ALS) [22].

Multiple phosphorylated tau (p-tau) isoforms—such as p-tau181, p-tau217, and p-tau 231—are characterized by distinct phosphorylation sites, each exhibiting unique clinical implications as blood-based biomarkers in AD and other neurodegenerative disorders [29]. Among these, p-tau181 has been most extensively validated and is now considered an established biomarker in clinical and research settings. Recent advances in automated immunoassays have enabled its robust quantification, with plasma concentrations showing a strong correlation with CSF levels. p-tau 217 has recently emerged as a highly specific indicator of cerebral amyloid pathology, rising at earlier stages along the AD continuum and offering superior diagnostic specificity for AD. p-tau 231 appears to increase even earlier—shortly after Aβ deposition—and is being investigated as a potential biomarker for detecting preclinical AD. Notably, elevated p-tau181 and p-tau 217 levels have also been reported in ALS [30,31]. One report has raised the possibility that a portion may originate from skeletal muscle rather than the central nervous system [31]. Thus, in myotonic dystrophy (DM)—a skeletal muscle disorder accompanied by CNS involvement but lacking amyloid pathology—examining alterations in p-tau181 or p-tau 217, which is relatively independent of amyloid-related changes, may provide valuable insights into disease mechanisms and neurodegenerative processes distinct from those observed in AD.

Although CSF and plasma biomarkers have also been explored in DM1, previous studies have been limited by small sample sizes or by the narrow range of biomarkers examined [32,33,34,35,36,37,38]. Given the difficulty of objectively quantifying CNS dysfunction in DM1, there is a clear need for reliable, quantifiable indicators that can sensitively capture the underlying neuropathological processes. In this exploratory study, we investigated four well-established biomarkers reflecting CNS injury and neurodegeneration-amyloid beta, GFAP, NfL, and p-tau181- in a cohort of patients with DM1, aiming to provide a more comprehensive preliminary characterization of CNS involvement in this multisystem disorder.

## 2. Materials and Methods

### 2.1. Study Participants

We recruited patients with DM1 from the NHO Aomori National Hospital, NHO Akita National Hospital, NHO Asahikawa National Hospital, NHO Suzuka National Hospital, Yokohama Rosai Hospital, and the University of Osaka Hospital. Informed consent was obtained from all participants with DM1. For healthy controls, we used previously reported data from participants at Fujita Health University [39]. All procedures involving human participants were performed in accordance with the ethical standards of the 1964 Helsinki Declaration and its later amendments. This study was approved by the Osaka University Clinical Research Review Committee [approval no. 20482(T2)-3].

Patients with DM1 were evaluated for the following clinical and genetic characteristics: age, age of onset for muscle symptom, sex, years of education, cognitive function assessed using the Mini-Mental State Examination (MMSE), activities of daily living assessed using the modified Rankin Scale (mRS), serum creatine kinase (CK), medical history including diabetes, ventilator use, and implantation of a pacemaker or implantable cardioverter defibrillator. The CTG repeat number of the *DMPK* gene, measured by Southern blotting, was obtained from medical records.

Healthy controls were obtained from participants who met the following criteria: (1) cognitively normal with MMSE scores greater than 25 and an Addenbrooke’s Cognitive Examination-Revised total score greater than 88 without a history of neurological or psychiatric disorders and (2) no observable anatomical abnormality in the brain according to magnetic resonance imaging [39].

### 2.2. Measurement of Blood Biomarkers

Blood samples were collected after >6 h of fasting. The samples were centrifuged for 10 min at 1500× *g*, and 500 μL aliquots of plasma were immediately frozen and stored at −80 °C until assayed. The plasma GFAP, NfL, Aβ40, Aβ42, and p-tau181 levels were determined with a single-molecule array (Simoa) using the Simoa Human Neurology 4-Plex E kit and the Simoa pTau-181 V2 Advantage kit (Quanterix, Billerica, MA, USA), according to the manufacturer’s protocol. Detailed information, such as cut-off, limit of detection, and lower limit of quantification, can be found in the manufacturer’s datasheet [40,41]. Plasma samples were tested in duplicates and each plate included in-house pooled controls. Each assay run included a multi-point calibration curve prepared from kit-supplied reference calibrators (A–G for pTau-181 V2; A–F for Neurology 4-Plex E). Calibrators were serially diluted in the provided diluent and measured in duplicate. Data were fitted with a four-parameter logistic (4PL) model using 1/y^2^ weighting.

Normal cut-off values for each plasma biomarker were referenced to existing in-house data, as follows: Aβ42/40: 0.05553; p-tau181: 2.294; NfL: 21.35; and GFAP: 249.85.

### 2.3. Statistical Analysis

Statistical analyses were performed using JMP software version 18 (JMP Statistical Discovery, Cary, NC, USA). The Mann–Whitney test was used to examine differences between the control and DM1 groups, as well as between males and females. The proportions exceeding the cut-off were compared between the two groups using the chi-square test. For the four biomarker values, a rank analysis of covariance (ANCOVA) was performed with age as a covariate. Spearman’s correlation coefficient was calculated to assess the correlations. Statistical significance was set at less than 0.05. To compare the three groups based on their MMSE scores, the Jonckheere-Terpstra trend test was performed.

## 3. Results

### 3.1. Participant Characteristics

Forty patients with DM1 and 38 healthy elderly controls were recruited for this study. Demographic information for the patients and control groups, as well as the patients’ clinical characteristics, is presented in Table 1. Patients with DM1 were approximately 20 years younger and had significantly fewer years of education compared with the control group. Although MMSE scores were similar between the two groups, a slight but statistically significant difference was observed (Table 1). In the control group, impaired glucose tolerance was observed in 13.1% of participants, and approximately one-fourth had hypertension and/or dyslipidemia. Data on renal dysfunction were not available, and information on hypertension or dyslipidemia was not collected for patients with DM1.

### 3.2. Comparison of Biomarker Levels Between DM1 Patients and Controls

**Figure 1:**
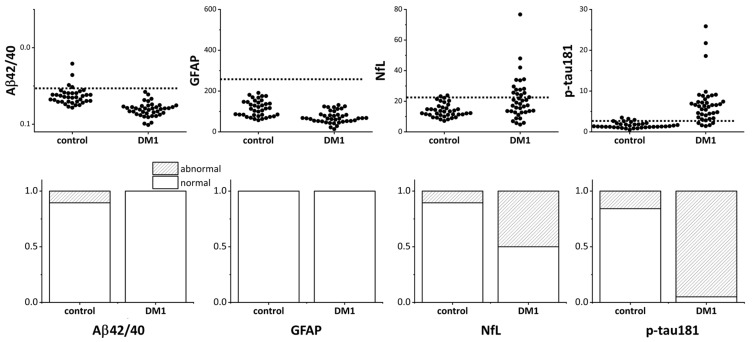
Plasma CNS biomarkers in patients with DM1 and healthy elderly controls. The top panel shows the values of plasma Aβ42/Aβ40, GFAP, NfL, and p-tau181 measured in patients with DM1 and healthy controls. The dotted line in each graph indicates the normal cutoff level. Because the Aβ42/Aβ40 ratio decreases in Alzheimer’s disease, the y-axis of the upper left Aβ42/Aβ40 graph is inverted so that smaller values are displayed upward. In the bottom panel, the shaded area indicates the portion that exceeded the normal cutoff level. CNS: central nervous system; DM1: myotonic dystrophy; Aβ: amyloid β, GFAP: glial fibrillary acidic protein; NfL: neurofilament light chain; p-Tau: phosphorylated tau.

For Aβ, the Aβ42/Aβ40 ratio—which typically decreases in Alzheimer’s disease—was calculated. Plasma CNS biomarkers, including Aβ42/Aβ40, GFAP, NfL, and p-tau181, in patients with DM1 and healthy elderly controls are shown in Figure 1. The percentage of patients with DM1 exceeding the normal cut-off obtained in a previous study was significantly higher for NfL and p-tau181, 50% and 95%, respectively. Notably, almost all patients with DM1 had p-tau181 values exceeding the cutoff (Figure 1, bottom).

None of the patients had abnormal Aβ42/Aβ40 and GFAP values, as judged by the cutoff values. These results differ significantly from our control subjects, in which NfL and p-tau181 were elevated in 10.5% and 15.8% of the subjects, respectively.

To compare the four biomarker values between the DM1 and control groups, rank ANCOVA was performed with age as a covariate, as the patients with DM1 were, on average, 20 years younger than the healthy controls. After age adjustment, NfL, p-tau, and Aβ42/40 showed significant group differences, although the effect size (F value) was smaller for Aβ42/40 (Table 2). GFAP levels did not differ between the groups.

### 3.3. Sex-Related Difference in Biomarkers

Subsequently, we examined sex-related differences. Previous studies have reported that male patients with DM1 tend to exhibit more pronounced central nervous system involvement [42,43]. In addition, investigations of CNS biomarkers across the AD continuum have demonstrated sex-related differences, with higher levels of GFAP-and possibly p-tau-observed in women [44,45].

In our cohort, women in the control group were significantly younger than men but showed higher GFAP levels and lower NfL levels (Table 3). No significant sex differences were observed in Aβ42/40 or p-tau181. In contrast, among patients with DM1, men were significantly younger than women but had lower MMSE scores and higher p-tau181 concentrations, while no significant sex differences were observed in the other three biomarkers.

### 3.4. Correlation Analyses Among Biomarkers and Clinical Parameters in DM1

**Figure 2:**
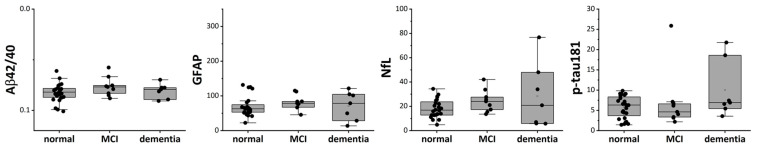
Plasma CNS biomarkers in patients with DM1 classified using MMSE. CNS plasma biomarker levels were shown when patients with DM1 were classified into three groups based on MMSE scores: normal, MCI dementia, and dementia. DM1: myotonic dystrophy; MCI: mild cognitive impairment; Aβ: amyloid β, GFAP: glial fibrillary acidic protein; NfL: neurofilament light chain; p-Tau: phosphorylated tau.

To examine the associations between each biomarker and cognitive function, patients with DM1 were classified into three categories according to their MMSE scores: normal (28–30), mild cognitive impairment (MCI) (24–27), and dementia (23 or below). The relationships between biomarker levels and these cognitive categories were then evaluated. Although no statistically significant differences were observed, the NfL and p-tau181 levels tended to be higher in the dementia group (Figure 2 and Supplemental Figure S1).

When the correlations between the four biomarkers and age, age at onset, CTG repeat length, mRS, and CK were examined, GFAP and NfL were found to be moderately correlated with patient age and age at onset, and NfL was correlated with mRS moderately but relatively modest in magnitude (Table 4). Interestingly, p-tau181 correlated with CK (Table 3 and Supplemental Figure S1). The disease duration, defined as the current age minus the age at onset, showed no significant correlation with any of the four biomarkers.

Furthermore, when correlations between biomarkers were examined, a significant correlation was found between NfL and GFAP and Aβ42/40. In contrast, no significant correlation was found between p-tau181 and the other biomarkers (Supplementary Table S1).

## 4. Discussion

CNS involvement is well recognized as a major contributor to the disease burden in DM1; however, its objective and quantitative assessment remains challenging. Conventional approaches such as neuroimaging or neuropsychological testing are often limited by feasibility and sensitivity for longitudinal evaluation. In this context, blood-based biomarkers that reflect neuroaxonal and glial pathology offer a promising means to capture CNS alterations in DM1. In the present study, we demonstrated significant elevations of plasma NfL and phosphorylated tau at threonine 181 (p-tau181) in patients with DM1, supporting their potential as circulating indicators of CNS pathology. NfL moderately correlated with age and functional severity, whereas p-tau181 exhibited a distinct elevation pattern, suggesting additional disease-related mechanisms. Collectively, these findings underscore the promise of CNS-derived plasma biomarkers for objective disease monitoring and therapeutic evaluation in DM1.

### 4.1. NfL

NfL is a subunit of the intermediate filament that forms the structural backbone of axons. It is released into the extracellular space following axonal injury. NfL reflects axonal degeneration and neuronal loss caused by neurodegenerative, demyelinating, or traumatic processes [28]. Although it lacks disease specificity, it serves as a sensitive biomarker for monitoring disease activity, progression, and therapeutic response.

It has been previously reported that blood NfL levels are elevated in patients with DM and correlate with age and CTG repeat length; findings that are largely consistent with those of this study [32,33,34,37]. Although based on limited evidence, there have been reports suggesting markedly elevated NfL levels in congenital and childhood-onset DM1 [33]. Consistent with these earlier findings, a few patients with relatively early-onset disease in our cohort also exhibited notably high NfL concentrations. While no statistical correlation with the MMSE was observed, a significant but relatively modest in magnitude correlation with activities of daily living (the mRS) was identified. This highlights the need to investigate whether blood NfL levels are associated with motor function. In DM1, NfL has generally been regarded as a blood biomarker that quantitatively reflects the extent of axonal degeneration, predominantly of central nervous system origin. DM1 is a multisystem disorder affecting the peripheral nerves, skeletal muscle, and central nervous system, and peripheral neuropathy is relatively common, although typically mild in degree. As the elevation of NfL in ALS is considered to derive mainly from axonal injury of motor neurons, it remains to be elucidated to what extent peripheral nerve involvement contributes to the increased NfL levels observed in DM1. Further investigation into the origin of circulating NfL in this disease is warranted.

### 4.2. p-Tau181

This study demonstrated for the first time that plasma p-tau181 levels were significantly elevated in patients with DM1, suggesting the potential of this biomarker. While total tau has been previously studied, the findings have varied, with some reports indicating an increase in CSF [32,35] and others reporting a decrease in blood [34]. p-tau181 has been detected in CSF but not in blood. Although earlier studies reported no significant changes [35,36], a recent study revealed a slight elevation in CSF p-tau181 in a small subset of patients with DM with cognitive decline compared to normal controls [32].

Elevated p-tau181 levels are believed to reflect amyloid pathology in AD [26]. Interestingly, this study found no correlation between plasma p-tau181 and the Aβ42/40 ratio in patients with DM1. Instead, a trend suggested an inverse relationship. Additionally, although not statistically significant, plasma p-tau181 levels negatively correlated with age, with younger patients exhibiting particularly high values. These findings suggest that the observed increase in p-tau181 levels in patients with DM1 is not linked to amyloid pathology.

Recent studies have demonstrated elevated serum p-tau181 and p-tau217 levels in patients with ALS, possibly originating from skeletal muscle [31]. Similar to the reported correlation between serum p-tau181 and serum troponin levels in patients with ALS, the present study revealed a correlation between plasma p-tau181 and serum CK. Further research is required to clarify the underlying mechanisms and pathological implications of elevated plasma p-tau181 in patients with DM1.

This study represents the first investigation of plasma p-tau181 in patients with DM1. Considering the likelihood that circulating p-tau181 concentrations in this population would be extremely low, we employed the Simoa platform, which currently provides the highest analytical sensitivity among blood-based biomarker assays established for AD [24]. Our work therefore serves as an initial step toward the clinical application of plasma p-tau181 measurement in DM1. Future studies are warranted to validate these findings using alternative analytical platforms that, while offering slightly lower sensitivity, may provide more cost-effective and widely accessible options for clinical practice. Collectively, our results lay the groundwork for integrating ultrasensitive biomarker technologies into the study of non-amyloid neurodegenerative processes associated with DM1.

### 4.3. Limitations

This study had several limitations. First, the lack of age-matched control data is a significant constraint. Consequently, the Aβ ratio in the DM group appeared to be more favorable than that in the control group according to the rank ANCOVA, even after adjusting for age. Although using younger, age-matched controls might have accentuated group differences in NfL and p-tau181 levels, such a design would also have introduced substantial disparities in cognitive performance (MMSE scores), complicating interpretation. It is therefore noteworthy that significant biomarker differences were observed despite broadly comparable cognitive performance in this older control group.

Furthermore, cognitive function was assessed solely using the MMSE, and the small sample size limited our ability to effectively analyze correlations with MMSE scores. As described in the Introduction, we restricted our analysis to p-tau181, given its established clinical validation and broader availability, whereas p-tau217 and p-tau231 were not assessed in this study. Future studies should investigate the relationship between biomarkers, a broader range of neuropsychological assessments, and brain imaging. Additionally, longitudinal studies are required to establish whether these biomarkers reflect the clinical course over time.

## Figures and Tables

**Table 1:** The characteristics of the participants.

	DM1, n = 40	Control, n = 38	*p* Value
Age (years)	47.5 [39, 50]	68 [64, 73]	<0.0001
Sex (male)	45%	50%	0.83
Education (years)	12 [12, 14]	14 [12, 16]	0.008
MMSE	28.5 [25, 30]	29 [28, 30]	0.041
Age at onset (years)	28.5 [20, 39]		
CTG repeat	1500 [975, 2175]		
mRS	3 [2, 4]		
CK	195 [112, 291] (n = 38)		
Wheelchair	25.0%	0%	0.001
Impaired glucose tolerance	32.5%	13.2%	0.043
Pacemaker	7.5%	0%	0.158
Ventilator	12.5%	0%	0.010

Median [1st quartile, 3rd quartile] are presented. Statistical analyses were performed using the Mann–Whitney test to examine group differences, except for sex, which was analyzed using the chi-square test. MMSE: mini-mental state examination; mRS: modified Rankin scale.

**Table 2:** Comparison of four plasma biomarkers between DM1 and control by rank ANCOVA adjusting the age.

	DM (n = 40)	Control (n = 38)	F	*p* Value
Aβ42/40	0.0797 [0.0763–0.0865]	0.0648 [0.0593–0.0754]	16.4	<0.0001
GFAP	66.5 [53.7–85.4]	113.8 [81.2–146.7]	0.59	0.4450
NfL	18.7 [13.0–27.1]	12.8 [11.1–16.2]	45.4	<0.0001
p-tau-181	6.32 [3.70–7.85]	1.40 [1.18–1.91]	45.1	<0.0001

Median [1st quartile, 3rd quartile] is presented. ANCOVA: rank analysis of covariance; Aβ: amyloid β, GFAP: glial fibrillary acidic protein; NfL: neurofilament light chain; p-tau: phosphorylated tau.

**Table 3:** Sex-related difference in biomarkers in the patients with DM1 and control.

	Control			DM1		
	Male	Female	*p*-Value	Male	Female	*p*-Value
	n = 19	n = 19		n = 18	n = 22	
Age	70 [66.5–73.5]	66 [63–39]	0.016	41.5 [29.3–48]	50 [45.25–52.5]	0.005
Years of Education	16 [12–16]	14 [12–14]	0.019	12 [12–13.75]	12 [12–14]	0.271
MMSE	29 [28, 29]	29 [28.5–30]	0.142	26 [23.3–29]	29 [27.25–40]	0.043
Aβ42/40	0.064 [0.059–0.070]	0.064 [0.060–0.072]	0.290	0.079 [0.073–0.081]	0.083 [0.078–0.088]	0.050
GFAP	85.2 [76.7–120.8]	130.0 [113.8–146.9]	0.031	64.8 [54.3–76.1]	72.5 [53.1–97.4]	0.167
NfL	14.80 [11.95–19.15]	11.51 [10.24–13.93]	0.033	25.12 [12.90–32.68]	16.85 [13.52–21.63]	0.089
p-tau181	1.33 [1.16–1.70]	1.58 [1.26–2.01]	0.207	7.02 [5.16–8.71]	5.028 [3.21–6.73]	0.029

Median [1st quartile, 3rd quartile] is presented. Statistical analyses were performed using the Mann–Whitney test to examine group differences. MMSE: mini-mental state exam, Aβ: amyloid β, GFAP: glial fibrillary acidic protein; NfL: neurofilament light chain; p-tau: phosphorylated tau.

**Table 4:** Correlations between plasma biomarkers and clinical parameters.

	Age		Onset Age		mRS		CTG		CK	
	Rho	*p* Value	Rho	*p* Value	Rho	*p* Value	Rho	*p* Value	Rho	*p* Value
Aβ42/40	−0.0303	0.8525	−0.1774	0.2735	−0.0041	0.9801	−0.1813	0.2630	0.2943	0.7290
GFAP	0.5087	0.0008	0.3371	0.0334	0.2225	0.1675	0.1141	0.4834	−0.2666	0.1056
NfL	0.4370	0.0048	0.4382	0.0047	0.3968	0.0113	0.2728	0.0885	−0.2930	0.0742
p-tau181	−0.1337	0.4107	−0.2422	0.1321	0.1415	0.3837	0.0076	0.9629	0.5779	0.0001

Spearman’s correlation coefficients (rho) and *p*-values are shown. mRS: modified Rankin scale; Aβ: amyloid β, GFAP: glial fibrillary acidic protein; NfL: neurofilament light chain; pTau: phosphorylated tau.

## Data Availability

The datasets generated and/or analyzed during the current study are not publicly available because of privacy constraints related to ethical approval but are available from the corresponding author upon reasonable request.

## Supplementary Materials

The following supporting information can be downloaded at https://www.mdpi.com/article/10.3390/jcm14228197/s1, Table S1: The correlations within plasma biomarkers in DM1 patients; Figure S1: Correlation of p-tau181 with CK and MMSE.

## Abbreviations

The following abbreviations are used in this manuscript:

Aβ	amyloid beta
AD	Alzheimer’s disease
ALS	amyotrophic lateral sclerosis
ANCOVA	analysis of covariance
CK	creatine kinase
CNS	central nervous system
CSF	cerebrospinal fluid
DM	Myotonic dystrophy
GFAP	glial fibrillary acidic protein
MCI	mild cognitive impairment
MMSE	Mini-Mental State Examination
mRS	modified Rankin Scale
NfL	neurofilament light chain
pTau	phosphorylated tau
PSP	progressive supranuclear palsy
Simoa	single-molecule array
3R	three-repeat
4PL	four-parameter logistic
4R	four-repeat
