# Supplementary material for: Plasma Neurofilament Light Chain and Phosphorylated Tau Are Elevated in Myotonic Dystrophy Type 1
Source: J Clin Med. 2025 Nov 19;14(22):8197. doi: 10.3390/jcm14228197 (PMC12653599; doi:10.3390/jcm14228197)

Supplementary Table 1 The correlations within plasma biomarkers in DM1 patients

	Aβ 42/40		GFAP		NF-L		p-tau181	
	rho	(*p* value)	rho	(*p* value)	rho	(*p* value)	rho	(*p* value)
Aβ42/40	-	-	-0.2525	(0.1159)	**-0.4375**	(0.0048)	0.233	(0.1479)
GFAP			-	-	**0.4478**	(0.0038)	-0.0662	(0.6847)
NF-L					-	-	0.246	(0.126)
p-tau181							-	-

Supplementary Figure 1.　Correlation of p-tau181 with CK and MMSE

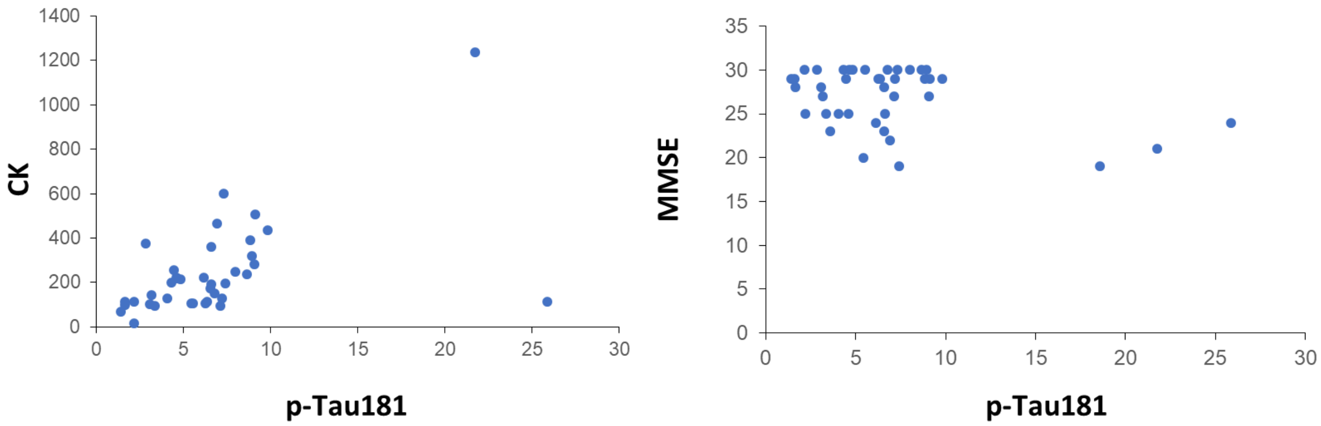

Supplement: Supplementary file 1 [file jcm-14-08197-s001.zip › jcm-3901664-supplementary.pdf]
